# Genomic Epidemiology of the First Wave of SARS-CoV-2 in Italy

**DOI:** 10.3390/v12121438

**Published:** 2020-12-14

**Authors:** Francesca Di Giallonardo, Sebastian Duchene, Ilaria Puglia, Valentina Curini, Francesca Profeta, Cesare Cammà, Maurilia Marcacci, Paolo Calistri, Edward C. Holmes, Alessio Lorusso

**Affiliations:** 1 The Kirby Institute, The University of New South Wales (UNSW), Sydney 2052, Australia; 2 Department of Microbiology and Immunology, The Peter Doherty Institute for Infection and Immunity, The University of Melbourne, Melbourne 3010, Australia; sebastian.duchene@unimelb.edu.au; 3 Istituto Zooprofilattico Sperimentale dell’Abruzzo e del Molise G. Caporale, 64100 Teramo, Italy; i.puglia@izs.it (I.P.); v.curini@izs.it (V.C.); f.profeta@izs.it (F.P.); c.camma@izs.it (C.C.); m.marcacci@izs.it (M.M.); p.calistri@izs.it (P.C.); a.lorusso@izs.it (A.L.); 4 Dipartimento di Medicina Veterinaria, Università degli Studi di Bari, 70010 Valenzano, Italy; 5 Marie Bashir Institute for Infectious Diseases and Biosecurity, School of Life & Environmental Sciences and School of Medical Sciences, The University of Sydney, Sydney 2006, Australia; edward.holmes@sydney.edu.au

**Keywords:** SARS-Cov-2, Italy, lockdown, phylogeny, transmission

## Abstract

Italy was one of the first countries to experience a major epidemic of severe acute respiratory syndrome coronavirus 2 (SARS-CoV-2), with >1000 cases confirmed by 1 March 2020. However, virus genome sequence data is sparse and there has been only limited investigation of virus transmission across the country. Here, we provide the most extensive study to date of the genomic epidemiology of SARS-CoV-2 in Italy covering the first wave of infection. We generated 191 new full-length genomes, largely sampled from central Italy (Abruzzo), before, during, and after the enforcement of a nationwide “lockdown” (8 March–3 June). These were combined with 460 published SARS-CoV-2 sequences sampled across Italy. Phylogenetic analysis including global sequence data revealed multiple independent introductions into Italy, with at least 124 instances of sequence clusters representing longer chains of transmission. Eighteen of these transmission clusters emerged before the nation-wide lockdown was implemented on 8 March, and an additional 18 had evidence for transmission between different Italian regions. Extended transmission periods between infections of up to 104 days were observed in five clusters. In addition, we found seven clusters that persisted throughout the lockdown period. Overall, we show how importations were an important driver of the first wave of SARS-CoV-2 in Italy.

## 1. Introduction

Eighteen years after the emergence of severe acute respiratory syndrome (SARS) in China (WHO) and eight years after the emergence of the Middle East respiratory syndrome (MERS) in Saudi Arabia [1], a novel coronavirus pandemic of animal origin emerged in late 2019 [2]. The disease, referred to as coronavirus disease 2019 (COVID-19), is caused by a novel *Betacoronavirus* named SARS coronavirus 2 (SARS-CoV-2) [3,4,5]. The virus was first identified in Wuhan, Hubei province, China, where pneumonia cases of unknown origin were observed in mid-December 2019 [6]. By the end of January 2020, about 20 other countries reported COVID-19 cases internationally, and at the time of writing, approximately 69 million cases of COVID-19 have been confirmed globally, with over 1.5 million deaths. Italy was the first European country to experience a major COVID-19 epidemic, with a first wave of transmission characterised by a relatively high number of deaths. As of 6 December 2020, 1,709,991 cases and 59,514 fatalities have been reported in Italy (WHO update, 8 December) [7].

**Figure 1:**
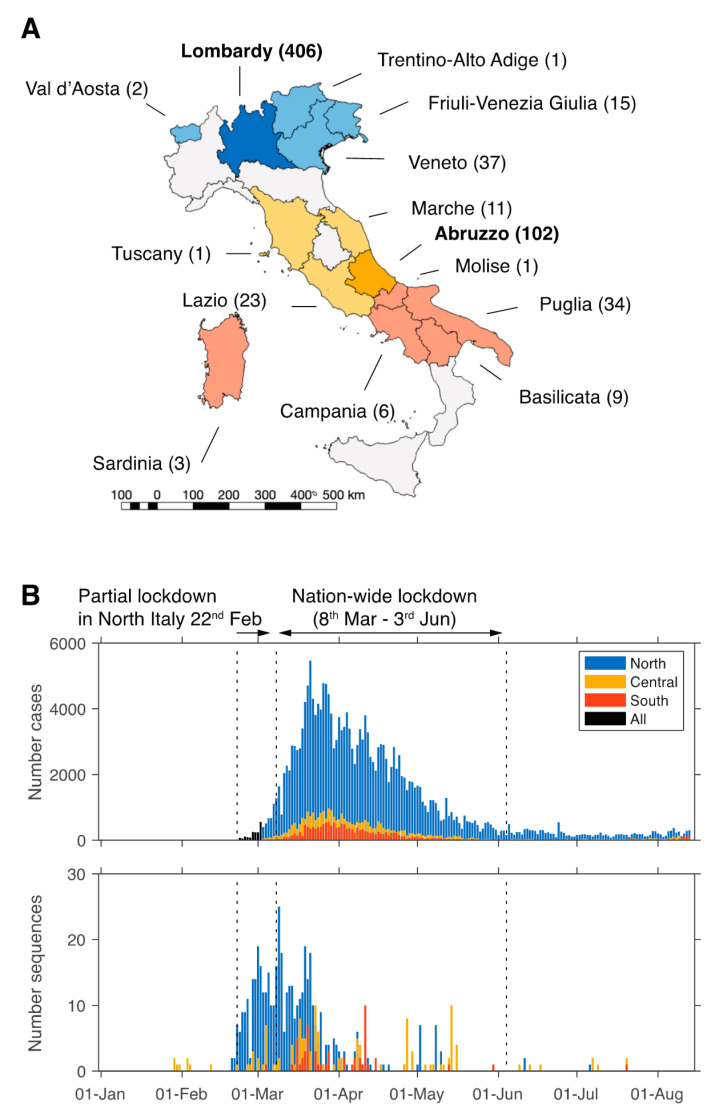
Number of COVID-19 cases and severe acute respiratory syndrome coronavirus 2 (SARS-CoV-2) genomes sequences per region and over time in Italy. (**A**) Italian regions with virus sequence data are coloured, and name of the geographic region and number of sequences are indicated. (**B**) The number of cases (top)—extracted from publicly available database [11]—and sequences (bottom) for the North, Central, and South regions is shown over time. The period of lockdown is indicated. Blue = North Italy, yellow = Central Italy, orange = South Italy, black = not specified.

The first confirmed cases of COVID-19 in Italy were reported on 30 January 2020 in Chinese tourists in Rome. This rose to nine confirmed cases by 22 February (WHO situation report 33) and 76 by 23 February (WHO situation report 34) [8,9]. Due to this alarming rise, the Italian government imposed a partial lockdown across 11 municipalities on February 22 in the two hotspot areas—Lombardy and Veneto [10]. This consisted of restrictions on individual mobility, social distancing, and closure of all non-essential services. Nevertheless, the number of confirmed cases rose exponentially, with 1128 total cases confirmed on 1 March. In response to this growing number of cases, the emergency lockdown measures were extended to the whole country on March 8. Notably, the majority of cases occurred in northern Italy in the region of Lombardy (Figure 1).

Previous studies have reported that the first cases reported in Lombardy were not related to those detected in Rome at the end of January, but rather derived from infections linked to Shanghai, China and Munich, Germany [12]. SARS-Cov-2 has been divided into two main lineages, A and B, each containing a number of sub-lineages [13]. Both lineages likely separated early during the Wuhan outbreak, with lineage B now being more widely distributed. A study investigating cases in the Lazio region (around Rome) reported that the majority of the analysed sequences belonged to the B.1 lineage, and dated the origin of the Italian epidemic to early February [10]. SARS-CoV-2 lineage B.1 contains an amino acid substitution at site 614 of the spike protein (D614G) that increases infectivity in cell assays in vitro and is proposed to enhance viral transmissibility in nature [14,15]. The earliest sequence detected carrying this genetic variation was sampled in Italy on 20 February, and became more common throughout Europe shortly after [16].

Using genomic epidemiology, we aimed to track the spread of SARS-CoV-2 in time and space within and between geographic regions in Italy during the first epidemic wave (between January to July 2020), focusing on the diversity of viral lineages present in the country before and after lockdown was imposed on 8 March 2020, and how they spread around the country, particularly the connections to the main disease hub in Lombardy. We focused on the central Italian region of Abruzzo for which we conducted virus sequencing. In Abruzzo, the first case of COVID-19 was recorded on 27 February, in a male patient who travelled as a tourist from Lombardy and arrived in Abruzzo shortly before the nation-wide lockdown was implemented across Italy. As per 6 December, 30,274 COVID-19 cases had been confirmed in Abruzzo, representing <2% of cases in Italy [17].

## 2. Materials and Methods

### 2.1. Ethics

The testing and sequencing of suspected COVID-19 cases and contacts in the Abruzzo region was conducted within the official surveillance program established by the Italian health authorities, and is exempt from ethical approval.

### 2.2. Specimen Collection

Samples were collected from the respiratory tract of individuals who were either hospitalized, screened through contact-tracing purposes, or tested via the framework of the screening programs for individuals working at the national health care system (Servizio Sanitario Nazionale, SSN). Samples were collected across Abruzzo in Teramo, Atri, Pescara, Avezzano, Sulmona, Lanciano, Castel di Sangro, Vasto, Chieti, and L’Aquila. In addition, swab specimens (*n* = 6843) were obtained from patients in Lombardy. SARS-Cov-2 RNA was confirmed as previously described [18]. Overall, 191 SARS-CoV-2-positive swab samples were sequenced.

### 2.3. Virus Genome Sequencing

RNA purified from positive samples of SARS-CoV-2 selected at the Istituto Zooprofilattico Sperimentale dell’Abruzzo e del Molise were processed for NGS by means of several approaches, including a combination of the sequence-independent, single-primer amplification (SISPA), and Nextera DNA Flex Library Prep (Illumina Inc., San Diego, CA, USA), as recently described [18]. Some of these libraries were enriched using a myBaits Expert Virus—SARS-CoV-2 kit (Arbor Biosciences, Ann Arbor, MI, USA). Targeted whole-genome amplification of SARS-Cov-2 approaches were also used, including the ARTIC amplicon sequencing protocol [19] and the Swift Amplicon^®^ SARS-CoV-2 Panel kit (Swift Biosciences, Ann Arbor, MI, USA). Deep sequencing was performed on the MiniSeq (Illumina Inc.) by the MiniSeq Mid Output Kit (300-cycles) and standard 150 bp paired-end reads.

### 2.4. Global SARS-CoV-2 Data Set

All available SARS-CoV-2 genomes from Italy derived by other research groups were downloaded from the GISAID EpiCov^TM^ database (13 August 2020, acknowledgment Table S4) and combined with the sequences obtained in the study here to form an Italian data set for further analysis. Because of the major sampling biases between individual regions, we combined genome sequences into larger geographical clusters: North Italy (71% of sequences; representing the Friuli Venezia Giulia, Lombardy, Trentino Alto Adige, Val D’Aosta and Veneto regions), Central Italy (21% of sequences; from Abruzzo, Lazio, Marche, Tuscany), and South Italy (8% of sequences; from Basilicata, Campania, Molise, Puglia, Sardinia).

To place the Italian sequences in the context of the global COVID-19 pandemic, they were compared against all available non-Italian SARS-CoV-2 genomes using Blastn. The top 50 hits for each Italian sequence were extracted in addition to 500 randomly selected global sequences and two early Wuhan sequences representative of viral clades A and B [13]—GISAID sequences hCoV-19/Wuhan/WH04/2020|EPI_ISL_406801|2020-01-05 and hCoV-19/Wuhan-Hu-1/2019|EPI_ISL_402125|2019-12-31 (Sequence IDs for all global sequences are provided in Table S1). This enabled us to obtain sufficiently informative sequences for phylogenetic analysis without generating a data set so large to be computationally intractable.

### 2.5. Phylogenetic Analysis

SARS-CoV-2 sequences were aligned using MAFFT implementing the L-INS-I algorithm, and the alignment was manually inspected in Geneious 11.1.3 (https://www.geneious.com). Sequences that were shorter than 95% of the complete genome, missing the day of sampling, contained at least 5% of ambiguous nucleotides, or that which displayed abnormally long branch lengths were excluded. Identical global sequences were also excluded. Nucleotide positions 6867 and 6868 (according to SARS-Cov-2 isolate Whuhan-Hu-1, NCBI accession MN908947) were removed from the alignment due to a potential sequencing error in some of the samples processed here. Accordingly, the final data set comprised 2373 sequences; 1722 global sequences, 651 sequences from Italy, of which 192 were created in this study here, and was 29,569 nucleotides in length. The sequences from Italy were sampled between 29th January to 20th July 2020, whereas the global sequences were sampled between 30th December 2019 and 29th July 2020.

SARS-CoV-2 were classified using the Pangolin COVID-19 Lineage Assigner tool v2.0.7 (https://pangolin.cog-uk.io/). A maximum likelihood tree of these data was estimated using IQ-TREE v1.6.12 [20], as described previously [21] using the Hasegawa–Kishino–Yano nucleotide substitution model with a gamma distributed rate variation among sites (HKY+Γ) [22], an ultrafast bootstrap method (1000 repetitions) and a minimum branch length for optimization at -blmin 1 × 10^−10^ nucleotide substitutions per site (subs/site). The tree was rooted between lineages A and B. A time tree was estimated using the maximum likelihood IQ-TREE approach, implementing a least-square dating algorithm (LSD) [23] with a fixed evolutionary rate of 8 × 10^−4^ subs/site, as estimated previously [21], and 1000 parametric bootstrap replicates to obtain confidence intervals in node ages. The genome sequence hCoV-19/Wuhan/WH04/2020|EPI_ISL_406801|2020-01-05 was used as an outgroup, as it falls in a basal position with respect to lineages A and B and it results in a reasonable estimate of the time of emergence (time to the most recent common ancestor, tMRCA). The tMRCA for the global data was between mid-November to late December, which is consistent with other studies and the first reported cases in Wuhan, China [2,24,25]. Only clusters with sufficient branch support (SH-aLTR >0.9 or bootstrap >70%) were considered for their tMRCA [26]. Importantly, we validated our estimates of node ages by analysing a subset of the data in BEAST 1.10 [27]. In this case, we used the same substitution model and outgroup as in the maximum likelihood analyses. We chose an exponential coalescent tree prior, with priors on population size and growth rate as in [24], and an uncorrelated relaxed molecular clock model with an underlying lognormal distribution. The exponential coalescent tree prior matches the expectation that the number of infected individuals overall was growing exponentially, and our choice of the relaxed molecular clock is based on previous studies that found strong statistical support for this model using Bayes factors [24]. To make our analyses comparable to those in LSD, we fixed the mean rate of to the same value used in LSD, while allowing rate variation among branches. We ran a Markov chain Monte Carlo of 5 × 10^−8^ steps sampling every 5000 steps, and we determined sufficient sampling from the posterior by verifying that all parameters had effective samples sizes of at least 500. Finally, all trees were visualised in FigTree v1.4.4.

### 2.6. Data Availability

Sequences are available via the GISAID EpiCov^TM^ database.

## 3. Results

### 3.1. Limited Availability of SARS-CoV-2 from Italy

The Italian Ministry of Health appointed the Istituti Zooprofilattici Sperimentali (IZSs), that comprises public veterinary institutes, to support the national health care system in conducting SARS-CoV-2 testing of rhino-pharyngeal swabs. The IZS responsible for the Abruzzo and Molise regions, the IZSAM, started testing human samples from Abruzzo, Molise, and southern regions on 16 March [18]. A total of 191 viral genomes covering the time period around 29 January to 20 July 2020 have successfully been sequenced and are included here. An additional 460 Italian genome sequences of SARS-CoV-2 available on GISAID were also included in the analysis. Hence, compared to the UK, from which over 118,000 genomes have been obtained, the genomic data from Italy is extremely limited, and represents only <0.1% of confirmed COVID-19 cases.

Overall, 62% of all Italian sequences were from Lombardy (*n* = 406), while 16% (*n* = 102) were from Abruzzo (Table S1). The remaining sequences were scattered across other Italian regions (Figure 1). Sequence data were available for the dates between 29 January–20 July 2020 and overall, the time-span of virus sampling was 173 days for Central Italy (29 January–20 July), 137 days for North Italy (2 February–6 July), and 138 days for South Italy (4 March–20 July).

### 3.2. Origin and Spread of SARS-CoV-2 in Italy

Sequence data were available before, during, and after the lockdown periods were imposed during the first wave in Italy. Overall, 12 sequences were sampled before any lockdown restrictions were imposed, and 184 sequences were sampled during the partial lockdown, of which 17 were from infections in Central Italy and only one from South Italy. Hence, the majority of the data (68%) was sampled during the nation-wide lockdown (*n* = 443), but only 24% (*n* = 104) and 12% (*n* = 51) were from Central and South Italy, respectively. Lastly, 12 sequences were available that were sampled after the lockdown restrictions were eased on 3 June. Eight of these (67%) were from infections in Central Italy (Abruzzo).

**Figure 2:**
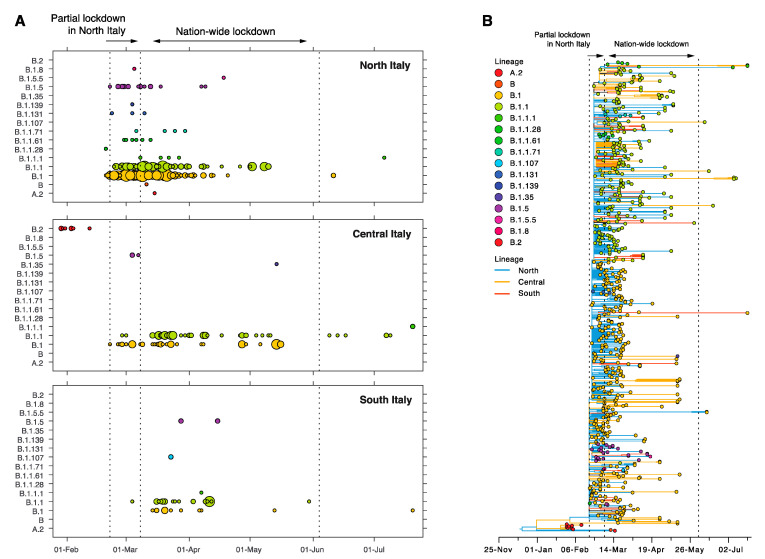
Distribution of SARS-CoV-2 lineages in Italy. (**A**) Number of sequences from different lineages across time for the three major geographic regions in Italy. The circle colour corresponds to each lineage and their size is proportional to the number of sequences sampled for a day for each lineage. Smallest circle = 1 sequence, largest circle = 15 sequences. (**B**) Italian sequences were extracted from a time-scaled tree (the global tree is shown in Figure S1). Branch lengths indicate the number of nucleotide substitutions per site and branches are coloured according to region; blue = North Italy, yellow = Central Italy, red = South Italy. Tip circles are coloured according to lineage.

Phylogenetic analysis revealed that all but one Italian sequence fell into SARS-CoV-2 lineage B: 50% were classified as lineage B.1 and 40% as B.1.1. Of the remaining sequences, 4% were B.1.5 and another 4% were a mix of numerous other B lineages (Figure 2A). One sequence was classified as A.2. This was isolated from an infection reported in Lombardy, although no epidemiological data or travel history was available via GISAID (EPI_ISL_542346). One B.2 sequence was sampled as the first locally acquired case in Rome (GISAID EPI_ISL_412974) and the remaining seven B.2 sequences were all from tourists who travelled from Hubei in China and visited Rome [28]. These infections have been extensively discussed elsewhere [12].

Next, we estimated the time-scale of the Italian epidemic. For lineage B.1, the 95% confidence interval of the tMRCA was estimated to be between 15–30 January 2020—for lineage B.1.1 it was 17–24 February, for lineage B.1.1.1 it was 21 February–14 March 2020, and finally, for B.1.5, we estimated it to be 13–21 February (Figure 2B). Thus, all lineages were estimated to have originated before the nation-wide lockdown was imposed in Italy. Of note, these overly precise estimates likely occurred because of the very large number of zero-length branches. Four sequences within the B.1 lineages were sampled before the partial lockdown in the North was imposed in Italy on 22 February, all from Lombardy. Eight different viral lineages were represented in the 184 sequences sampled during the partial lockdown (22 February–7 March), 11 lineages were present in the 442 sequences sampled during the nation-wide lockdown (8 March–5 June, excluding the one A.2 infection), and three lineages in the 12 sequences sampled after the lockdown restrictions were eased (Figure 2B, Table S2). Interestingly, three lineages were identified during the nation-wide lockdown, although not before; B.1.107 (*n* = 2), B.1.35 (*n* = 1), and B.1.5.5 (*n* = 1). Lineage B.1.1.1 also appeared during the nation-wide lockdown and persisted throughout this period, with three sequences of this lineage sampled after the lockdown in July 2020.

### 3.3. Sustained Local Transmission during Lockdown Period

**Figure 3:**
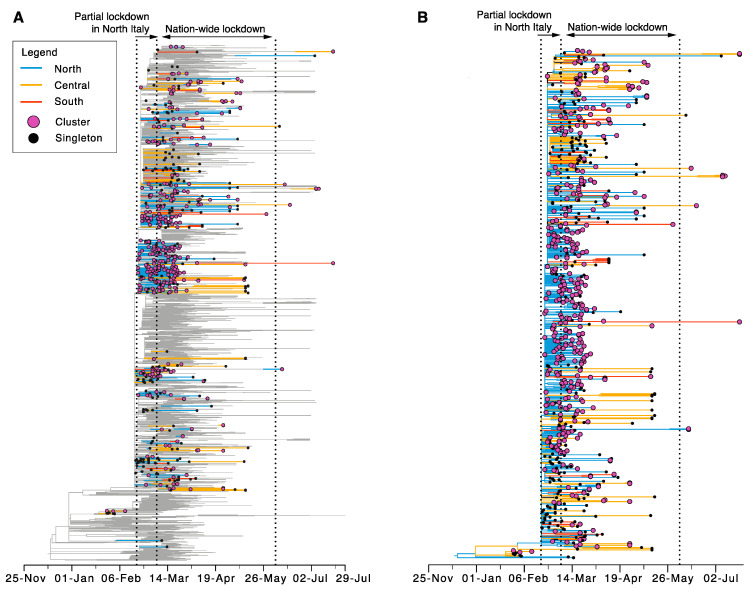
Identification of transmission clusters in Italy. The figures depict time-trees with branch lengths proportional to time in years. Branches are coloured according to geographic territory; blue = North Italy, yellow = Central Italy, red = South Italy. Tip circles are coloured according to clustering type; clusters = pink, singletons = black. Lockdown periods are marked by dotted lines. (**A**) Global tree, (**B**) Italian sequences extracted from global tree.

Overall, 124 independent Italian sequence clusters comprising 412 sequences were identified within the global phylogeny, and these likely represent individual introduction events into the country. The remaining 239 sequences (37%) were classified as “singletons”, as they were not related to any other Italian sequence and may again represent individual importation events into Italy (Figure 3). Similarly, singletons and sequence clusters from North, South, and Central Italy were scattered across the global phylogeny, indicative of multiple independent introductions into these regions. In total, 80 transmission clusters contained only sequences from infections in North Italy, while 14 and 11 clusters were exclusively associated with SARS-CoV-2 infections in Central and South Italy, respectively. Three relatively large clusters (containing 24, 34, and 37 sequences) were present in North Italy, the larger two of which contained identical sequences sampled within 29 and 35 days of each other (cluster numbers 52 and 71, respectively).

In the case of North Italy, 11 transmission clusters were sampled during the partial lockdown, 30 during the nation-wide lockdown, and one after lockdown restrictions were eased. In addition, 40 clusters were sampled that continued from the partial to the nation-wide lockdown. For infections in Central Italy, 11 clusters were sampled during the nation-wide lockdown and three after the lockdown period. Finally, for South Italy, 10 clusters were sampled during the nation-wide lockdown, and one cluster continued through the post-lockdown period. An additional 18 transmission clusters contained viruses sampled from multiple geographic regions: North/Central *n* = 13, North/South *n* = 3, Central/South *n* = 2 (Figure 3). Two of these were sampled during the partial lockdown, 10 during the nation-wide lockdown, five continued from the partial to the nation-wide lockdown, and one cluster persistent from the partial to the post-lockdown period.

Numerous clusters had very short internal branches and very low branch support, indicative of rapid transmission within them but also phylogenetic uncertainty. However, 54 clusters were found with sufficient node support, in turn enabling estimates of tMRCAs (Table S3). For these transmission clusters, the earliest mean tMRCA was estimated for 22 February for North Italy, just prior to the start of the partial lockdown in this region. For Central Italy, the earliest tMRCA was 30 January 2020, representing the B.2 lineages, while the earliest B.1 transmission cluster had an estimated tMRCA for 3 March. For South Italy, the first transmission cluster was dated to a mean of 28 February 2020. Finally, one cluster contained viral sequences from different geographic regions with an estimated tMRCA of 28 February. The cluster included infections from North Italy, which were sampled during the partial and nation-wide lockdown, as well as one infection from Central Italy that was sampled on 3 June. Overall, 18 transmission clusters originated before the nation-wide lockdown (North = 14, Central = 2, South = 1, Mixed = 1), 34 during the lockdown (North = 19, Central = 7, South = 8), and two (both from Central Italy) emerged after the lockdown restrictions were eased.

### 3.4. Limited Genetic Diversity

As expected, SARS-CoV-2 exhibited low levels of genetic diversity, with mean pairwise similarities of 99.4% for the complete data set and 99.6% for the Italian sequences. All Italian sequences belonging to lineage B.1 were characterised by the known two substitutions in the ORF1ab polyprotein at nucleotide positions 3037 (synonymous C→T) and 14,408 (non-synonymous C→T, amino acid P→L), as well as the substitution in the S protein at position 23,403 (non-synonymous A→G, amino acid D→G (all substitutions are compared to reference sequence Wuhan-Hu-1, NCBI accession MN908947). The latter mutation corresponds to the D614G amino acid substitution in the S protein [14]. The B.1.1 lineage is characterised by two amino acid substitutions in the *n* protein at positions 203 and 204 (MN908947 nt position 28882) [10]; lineage B.1.1 contains a KR motif here, while the other lineages in the Italian data set contain RG (Figure S1). Eight sequences were classified as B.1.1.1. This lineage is more distinct with four unique mutations, of which three are found in the ORF1ab polyprotein at nucleotide position 4002 (non-synonymous C→T, amino acid T→I), position 10,097 (non-synonymous G→A, amino acid G→S), position 13,536 (synonymous C→T), and one in the S protein at position 23,731 (synonymous C→T).

## 4. Discussion

We presented a genomic snapshot of the Italian epidemic of SARS-CoV-2, from early infections in late January 2020 to the end of the first wave in July 2020, post-lockdown. In particular, we showed that the epidemic started via multiple introductions and the time-scale of these events, particularly that new transmission clusters may have initiated even during the period of nationwide lockdown.

Despite the limited sequence data available, we identified multiple transmission clusters of Italian sequences scattered across the SARS-CoV-2 phylogeny that are indicative of approximately 124 independent introductions into Italy from this limited sample size, including 14 independent introductions into Abruzzo. Similarly, the large number of phylogenetic singleton sequences are also likely indicative of multiple introduction events. Evidence of multiple introductions of SARS-CoV-2 into Italy were reported as early as March 2020 [28], with initial positive cases reported in Rome that were linked to Wuhan but that did not lead to further infections, with the Italian epidemic eventually triggered by a distinct clade with links to other European countries, most likely Germany [12]. This was supported by a subsequent study that showed multiple introductions into North and Central Italy followed by the emergence of clade B.1 [29]. According to our estimates, the tMRCA for the Italian B.1 lineages was between 15–30 January 2020.

The majority of the Italian sequences sampled to date represent lineages B.1 (50%) and B.1.1 (40%), with the remaining sequences covering 14 other lineages, most at low frequency. Indeed, 92% of all transmission clusters comprised lineages B.1 and B.1.1. Thus, the overall genetic diversity represented in the Italian sequence data is limited, and it is likely that the rapid and strict lockdown enforced in the country led to a marked genetic bottleneck and lineage extinction. Indeed, only three lineages were present overall after the lockdown restrictions were eased (B.1, B.1.1, and B.1.1.1), all of which were already present during the lockdown period. Notably, three large clusters (containing 24–37 sequences), all from Lombardy in North Italy, consisted of mainly identical sequences that were sampled within 35 days, implying large-scale local transmission. Similar limited genetic diversity has been described in Iceland with higher sequence coverage, with only seven lineages within their infected population which may reflect the rapid containment by the public health authorities [30]. In contrast, New Zealand reported over 30 lineages and strong evidence for infections linked to travelers rather than community transmission [21]. Although New Zealand similarly implemented a strict lockdown and limited travel to the islands, it is possible that the greater diversity of viruses reflects the fact that it received travelers, and their viruses, from diverse global locations, whereas the Italian outbreak was likely largely seeded from neighbouring European countries. This will need to be confirmed with more sequence data and individual linked travel histories.

We found numerous independent transmission clusters scattered across different geographic regions within Italy, suggesting that there were multiple entries of genetically similar lineages and that these were the dominant ones circulating in the source populations at the time early in the COVID-19 pandemic. Notably, we observed that transmission occurred throughout the lockdown period and between the North, Central, and South regions. Notably, for the cluster in Central Italy, the earliest infection was not sampled until 7 April, some 35 days after the estimated tMRCA date on 3 March. Thus, we likely missed infections linking the cluster to earlier transmission events. It is known from case reports that the first positive case in Abruzzo was sampled on 27 February among a tourist visiting from the Lombardy region. Unfortunately, no virus sequence is available for this case. It has also been reported that numerous residents returned to Central Italy from Lombardy and surrounding regions before the nation-wide lockdown was imposed, and we found multiple clusters containing infections from North and Central Italy, indicative of cross-region transmission between these localities (although only one had sufficient support to accurately estimate the tMRCA).

## 5. Conclusions

We reported on the limited but ongoing within-region transmission during the first wave of SARS-CoV-2 in Italy, that the epidemic was seeded by multiple introductions of similar virus lineages into the country, that viruses travelled from North to South on multiple occasions, and that sequences from infections sampled after the lockdown period was eased were sometimes linked to infections from during the lockdown. These features are indicative of ongoing transmission throughout the lockdown period, rather than re-introduction of novel lineages past lockdown. However, it is critical to acknowledge the extremely limited amount of genomic data from Italy compared to many other localities that clearly impacted the strength of the conclusions that can be drawn here. It is therefore vital that Italy build better structures for effective genomic epidemiology prior to any future major outbreaks of emerging infectious disease.

## Supplementary Materials

The following are available online at https://www.mdpi.com/1999-4915/12/12/1438/s1, Figure S1: Phylogenetic analysis of the Italian SARS-CoV-2 genome sequences in a global context. Table S1: Sequence data, Table S2: Number of sequences sampled across Italy, Table S3: Time to most recent common ancestor (tMRCA) estimates for clusters with sufficient branch support, Table S4: GISAID EpiCov^TM^ acknowledgment.
