# Supplementary material for: Genomic Epidemiology of the First Wave of SARS-CoV-2 in Italy
Source: Viruses. 2020 Dec 14;12(12):1438. doi: 10.3390/v12121438 (PMC7765063; doi:10.3390/v12121438)
Supplement: Supplementary file 1 [file viruses-12-01438-s001.zip › viruses-1022733 Figure S1 Tables S2 and S3.docx]

**Supplementary Table S2. Number of sequences sampled across Italy.**

| **Lineage** | **North** | **Central** | **South** |
| --- | --- | --- | --- |
| *Before any lockdown* | |  |  |
| B.1 | 3 | 0 | 0 |
| B.1.1.28 | 1 | 0 | 0 |
| B.2 | 0 | 8 | 0 |
| *During partial lockdown in North Italy (22 February - 7 March)* | | | |
| B.1 | 110 | 12 | 0 |
| B.1.1 | 37 | 2 | 1 |
| B.1.1.61 | 4 | 0 | 0 |
| B.1.1.71 | 1 | 0 | 0 |
| B.1.131 | 2 | 0 | 0 |
| B.1.139 | 1 | 0 | 0 |
| B.1.5 | 10 | 3 | 0 |
| B.1.8 | 1 | 0 | 0 |
| *Nation-wide lockdown (8 March - 5 June)* | | |  |
| A.2 | 1 | 0 | 0 |
| B | 1 | 0 | 0 |
| B.1 | 146 | 42 | 12 |
| B.1.1 | 121 | 61 | 32 |
| B.1.1.1 | 4 | 0 | 1 |
| B.1.1.61 | 2 | 0 | 0 |
| B.1.1.71 | 3 | 0 | 0 |
| B.1.107 | 0 | 0 | 2 |
| B.1.131 | 1 | 0 | 0 |
| B.1.35 | 0 | 1 | 0 |
| B.1.5 | 8 | 0 | 4 |
| B.1.5.5 | 1 | 0 | 0 |
| *After lockdown (6 June and after)* | | |  |
| B.1 | 2 | 0 | 1 |
| B.1.1 | 0 | 6 | 0 |
| B.1.1.1 | 1 | 2 | 0 |

**Supplementary Table S3. Time to most recent common ancestor (tMRCA) estimates for clusters with sufficient branch support.** Transmission clusters are ordered according to Italian region (north to south) and tMRCA (oldest to recent).

| **Lineage** | **Number of sequences in cluster** | **Earliest infection** | **Most recent infection** | **Days from earliest to most recent** | **SH aLTR / boostrap (%)** | **tMRCA** | **CI lower** | **CI higher** |
| --- | --- | --- | --- | --- | --- | --- | --- | --- |
| *North Italy* |  |  |  |  |  |  |  |  |
| B.1 | 4 | 23-Feb-20 | 31-Mar-20 | 37 | 84.8/99 | 22-Feb-20 | 17-Feb-20 | 22-Feb-20 |
| B.1.1.71 | 2 | 06-Mar-20 | 25-Mar-20 | 19 | 78.8/86 | 25-Feb-20 | 17-Feb-20 | 25-Feb-20 |
| B.1.1 | 3 | 10-Mar-20 | 21-Mar-20 | 11 | 91.3/100 | 25-Feb-20 | 17-Feb-20 | 25-Feb-20 |
| B.1 | 2 | 24-Feb-20 | 09-Mar-20 | 14 | 87.4/100 | 25-Feb-20 | 17-Feb-20 | 25-Feb-20 |
| B.1 | 2 | 18-Mar-20 | 22-Mar-20 | 4 | 78.4/100 | 25-Feb-20 | 17-Feb-20 | 10-Mar-20 |
| B.1.1 | 3 | 13-Mar-20 | 01-Apr-20 | 19 | 79.2/100 | 25-Feb-20 | 17-Feb-20 | 14-Mar-20 |
| B.1 | 2 | 01-Mar-20 | 30-Mar-20 | 29 | 92.2/92 | 28-Feb-20 | 17-Feb-20 | 28-Feb-20 |
| B.1 | 2 | 28-Feb-20 | 28-Feb-20 | 0 | 83.5/100 | 28-Feb-20 | 25-Feb-20 | 28-Feb-20 |
| B.1 | 4 | 11-Mar-20 | 03-Apr-20 | 23 | 89.4/72 | 03-Mar-20 | 17-Feb-20 | 10-Mar-20 |
| B.1 | 2 | 05-Mar-20 | 09-Mar-20 | 4 | 92.5/100 | 03-Mar-20 | 22-Feb-20 | 03-Mar-20 |
| B.1 | 2 | 02-Mar-20 | 04-Mar-20 | 2 | 94/100 | 03-Mar-20 | 22-Feb-20 | 03-Mar-20 |
| B.1.1 | 4 | 08-Mar-20 | 20-Mar-20 | 12 | 86.3/100 | 03-Mar-20 | 22-Feb-20 | 08-Mar-20 |
| B.1.1 | 2 | 02-Mar-20 | 03-Mar-20 | 1 | 85.3/100 | 03-Mar-20 | 28-Feb-20 | 03-Mar-20 |
| B.1 | 2 | 05-Mar-20 | 05-Mar-20 | 0 | 84.4/100 | 03-Mar-20 | 28-Feb-20 | 03-Mar-20 |
| B.1 | 6 | 16-Mar-20 | 22-Mar-20 | 6 | 85.4/100 | 08-Mar-20 | 22-Feb-20 | 14-Mar-20 |
| B.1 | 6 | 06-Mar-20 | 20-Mar-20 | 14 | 81/100 | 08-Mar-20 | 25-Feb-20 | 08-Mar-20 |
| B.1 | 4 | 06-Mar-20 | 19-Mar-20 | 13 | 85.6/100 | 08-Mar-20 | 25-Feb-20 | 08-Mar-20 |
| B.1.1 | 2 | 22-Mar-20 | 25-Mar-20 | 3 | 86.5/100 | 10-Mar-20 | 22-Feb-20 | 21-Mar-20 |
| B.1 | 2 | 16-Mar-20 | 23-Mar-20 | 7 | 90.4/100 | 14-Mar-20 | 03-Mar-20 | 14-Mar-20 |
| B.1.1 | 3 | 17-Mar-20 | 19-Mar-20 | 2 | 82.2/100 | 14-Mar-20 | 03-Mar-20 | 18-Mar-20 |
| B.1.1.1 | 3 | 18-Mar-20 | 27-Mar-20 | 9 | 87/100 | 18-Mar-20 | 08-Mar-20 | 18-Mar-20 |
| B.1.1 | 2 | 21-Mar-20 | 21-Mar-20 | 0 | 91.3/100 | 21-Mar-20 | 10-Mar-20 | 21-Mar-20 |
| B.1.1 | 2 | 21-Mar-20 | 21-Mar-20 | 0 | 89.9/100 | 21-Mar-20 | 10-Mar-20 | 21-Mar-20 |
| B.1.1 | 4 | 21-Mar-20 | 31-Mar-20 | 10 | 91.8/100 | 21-Mar-20 | 10-Mar-20 | 21-Mar-20 |
| B.1 | 2 | 27-Mar-20 | 27-Mar-20 | 0 | 85.2/100 | 21-Mar-20 | 10-Mar-20 | 25-Mar-20 |
| B.1.1 | 2 | 31-Mar-20 | 01-Apr-20 | 1 | 85.7/100 | 29-Mar-20 | 14-Mar-20 | 01-Apr-20 |
| B.1.1 | 2 | 07-Apr-20 | 17-Apr-20 | 10 | 91.9/100 | 01-Apr-20 | 10-Mar-20 | 08-Apr-20 |
| B.1 | 3 | 08-Apr-20 | 08-Apr-20 | 0 | 94.5/100 | 05-Apr-20 | 21-Mar-20 | 08-Apr-20 |
| B.1.1 | 2 | 07-Apr-20 | 26-Apr-20 | 19 | 96.4/100 | 08-Apr-20 | 18-Mar-20 | 08-Apr-20 |
| B.1.1 | 2 | 08-Apr-20 | 10-Apr-20 | 2 | 77.2/80 | 08-Apr-20 | 25-Mar-20 | 08-Apr-20 |
| B.1 | 2 | 12-Apr-20 | 12-Apr-20 | 0 | 98.4/100 | 12-Apr-20 | 29-Mar-20 | 12-Apr-20 |
| B.1.1 | 3 | 10-May-20 | 10-May-20 | 0 | 93.3/100 | 30-Apr-20 | 12-Apr-20 | 11-May-20 |
| B.1 | 2 | 11-Jun-20 | 11-Jun-20 | 0 | 98.7/100 | 26-May-20 | 04-May-20 | 10-Jun-20 |
| *Central Italy* |  |  |  |  |  |  |  |  |
| B.2 | 3 | 29-Jan-20 | 12-Feb-20 | 14 | 86.1/100 | 30-Jan-20 | 15-Jan-20 | 30-Jan-20 |
| B.1 | 3 | 07-Apr-20 | 27-Apr-20 | 20 | 76.4/73 | 03-Mar-20 | 14-Feb-20 | 25-Mar-20 |
| B.1 | 2 | 14-Mar-20 | 17-Mar-20 | 3 | 76.3/89 | 08-Mar-20 | 22-Feb-20 | 14-Mar-20 |
| B.1.1 | 2 | 20-Mar-20 | 20-Mar-20 | 0 | 91.7/100 | 14-Mar-20 | 03-Mar-20 | 21-Mar-20 |
| B.1 | 2 | 16-Mar-20 | 26-Mar-20 | 10 | 90.3/68 | 14-Mar-20 | 08-Mar-20 | 14-Mar-20 |
| B.1.1 | 7 | 17-Mar-20 | 04-May-20 | 48 | 93.4/95 | 18-Mar-20 | 25-Feb-20 | 18-Mar-20 |
| B.1.1 | 2 | 24-Mar-20 | 30-Mar-20 | 6 | 92.4/99 | 21-Mar-20 | 10-Mar-20 | 25-Mar-20 |
| B.1.1 | 2 | 07-May-20 | 10-May-20 | 3 | 80.2/100 | 12-Apr-20 | 21-Mar-20 | 04-May-20 |
| B.1 | 2 | 27-Apr-20 | 27-Apr-20 | 0 | 93.9/100 | 23-Apr-20 | 08-Apr-20 | 27-Apr-20 |
| B.1.1 | 3 | 07-Jul-20 | 09-Jul-20 | 2 | 98.3/100 | 17-Jun-20 | 22-May-20 | 05-Jul-20 |
| B.1.1.1 | 2 | 20-Jul-20 | 20-Jul-20 | 0 | 91.1/100 | 21-Jun-20 | 26-May-20 | 21-Jul-20 |
| *South Italy* |  |  |  |  |  |  |  |  |
| B.1 | 2 | 14-Mar-20 | 20-Mar-20 | 6 | 78.1/100 | 28-Feb-20 | 17-Feb-20 | 14-Mar-20 |
| B.1.1 | 2 | 20-Mar-20 | 09-Apr-20 | 20 | 86.8/86 | 14-Mar-20 | 28-Feb-20 | 21-Mar-20 |
| B.1.1 | 2 | 20-Mar-20 | 09-Apr-20 | 20 | 94.7/100 | 18-Mar-20 | 28-Feb-20 | 21-Mar-20 |
| B.1.1 | 2 | 18-Mar-20 | 19-Mar-20 | 1 | 79.3/100 | 18-Mar-20 | 08-Mar-20 | 18-Mar-20 |
| B.1 | 2 | 20-Mar-20 | 20-Mar-20 | 0 | 84.6/100 | 18-Mar-20 | 10-Mar-20 | 21-Mar-20 |
| B.1.5 | 4 | 28-Mar-20 | 15-Apr-20 | 18 | 93.6/100 | 21-Mar-20 | 28-Feb-20 | 29-Mar-20 |
| B.1.1 | 2 | 11-Apr-20 | 11-Apr-20 | 0 | 85.9/100 | 29-Mar-20 | 14-Mar-20 | 12-Apr-20 |
| B.1.1 | 2 | 11-Apr-20 | 11-Apr-20 | 0 | 86/100 | 01-Apr-20 | 14-Mar-20 | 12-Apr-20 |
| B.1 | 2 | 07-Apr-20 | 20-Jul-20 | 104 | 93.9/100 | 08-Apr-20 | 10-Mar-20 | 08-Apr-20 |
| *Mixed clusters* | |  |  |  |  |  |  |  |
| B.1 | 2 | 29-Feb-20 | 04-Mar-20 | 4 | 86.9/100 | 28-Feb-20 | 22-Feb-20 | 28-Feb-20 |

**Supplementary Figure S1. Phylogenetic analysis of the Italian SARS-CoV-2 genome sequences in a global context.** The branches are coloured according to lineage and Italian sequences are marked with a circle for North = blue, Central = yellow, and South = red. **(A)** Maximum likelihood tree with branch lengths in nucleotide substitutions per site. **(B)** Time-scaled tree with branch lengths proportional to x-axis.
